# Antimicrobial Resistance and Clinical Outcome Among Hospitalized Bacterial Pneumonia: A Retrospective Cohort Study in Indonesian Tertiary Hospital

**DOI:** 10.3390/antibiotics15060582

**Published:** 2026-06-08

**Authors:** Prayudi Santoso, Ghyna Ravifa Muliandini, Saniya Dhafarina Izzati, Sonya Alexandra, Iceu Dimas Kulsum, Basti Andriyoko, Adhi Kristianto Sugianli

**Affiliations:** 1 Department of Internal Medicine, Faculty of Medicine, Universitas Padjadjaran, Bandung 40161, Indonesia; 2 Department of Medicine, Dr. Hasan Sadikin General Hospital, Bandung 40161, Indonesia; 3 Faculty of Medicine, Universitas Padjadjaran, Bandung 40161, Indonesia; 4 Research Center for Care and Control of Infectious Disease (RC3ID), Universitas Padjadjaran, Bandung 40161, Indonesiaadhi.kristianto@unpad.ac.id (A.K.S.); 5 Department of Clinical Pathology, Faculty of Medicine, Universitas Padjadjaran, Bandung 40161, Indonesia; 6 Department of Supporting Medicine, Dr. Hasan Sadikin General Hospital, Bandung 40161, Indonesia

**Keywords:** community-acquired pneumonia, hospital-acquired pneumonia, ventilator-associated pneumonia, antimicrobial resistance, clinical outcome, Indonesia

## Abstract

**Background:** Pneumonia is a common cause of hospitalization and a significant contributor to worldwide morbidity and mortality. Effective definitive antimicrobial therapy for pneumonia relies on accurate identification of bacterial pathogens and their resistance patterns. Therefore, this study aims to evaluate the distribution bacterial pathogens and their antimicrobial resistance patterns, as well as clinical factors associated with outcomes among hospitalized pneumonia patients. **Methods:** This retrospective cohort study was conducted at Dr. Hasan Sadikin General Hospital, Indonesia, and included adult patients hospitalized with pneumonia between January and December 2024. Clinical, demographic, microbiological, and outcome data were extracted from electronic medical records and the laboratory system. Bacterial distribution, antimicrobial patterns, and clinical outcomes were analyzed descriptively and compared across pneumonia types. Multivariable regression analyses were performed to identify factors associated with in-hospital mortality and length of hospital stay. **Results:** A total of 662 hospitalized pneumonia patients were included with Gram-negative bacteria (i.e., *Klebsiella pneumonia*, *Acinetobacter baumannii*, and *Pseudomonas aeruginosa*) identified as the most common pathogens. Carbapenem-resistant *Acinetobacter baumannii* (CR-Ab) and *Klebsiella pneumoniae* (CR-Kp) were the most frequently identified resistant pathogens, particularly in hospital-acquired (HAP) and ventilator-associated pneumonia (VAP). HAP and VAP were independently associated with higher in-hospital mortality and longer hospital stay compared to community-acquired pneumonia (CAP). In addition, CR-Ab and difficult-to-treat *Pseudomonas aeruginosa* (DTR-Psa) were associated with prolonged hospitalization. **Conclusions:** Type of pneumonia, bacterial pathogens and resistance patterns were associated with in-hospital mortality and length of hospital stay. These findings highlight the importance of ongoing microbiological surveillance, antimicrobial stewardship, and infection prevention strategies to optimize pneumonia management and clinical outcomes.

## 1. Introduction

Antimicrobial resistance (AMR) is a major global health challenge and has been identified as one of the top 10 global public health threats by the World Health Organization (WHO) [1]. AMR compromises the effectiveness of antibiotics, resulting in more difficult-to-treat infections, increased disease severity, prolonged hospitalization, and higher mortality. Globally, approximately one in six laboratory-confirmed bacterial infections were caused by antibiotic-resistant bacteria, with a median resistance level of 17.2% across 93 monitored bacterial pathogen-antibiotics combinations [2]. In 2021, an estimated 4.71 million deaths were associated with bacterial AMR, of which 1.14 million were directly attributable to it [3].

Pneumonia is an inflammation of the pulmonary parenchyma, arising from the proliferation of microbial pathogens at the alveolar level and the host’s response to them [4]. Community-acquired pneumonia (CAP) refers to lung inflammation from infections acquired outside of a hospital setting, whereas pneumonia occurring >48 h after hospital admission is hospital-acquired pneumonia (HAP). Pneumonia that develops after ≥48 h of endotracheal intubation is ventilator-associated pneumonia (VAP) [5,6]. In the United States, the incidence of CAP requiring hospitalization was 649 cases per 100,000 adults, while in Europe, it varies across countries (50–2940 per 100,000 adults) [7,8]. The incidence of HAP in the United States is estimated to be 3.63 cases per 1000 patient-days, while the incidence of VAP is at 13.5 cases per 1000 ventilator-days [9,10]. In Asia, the incidence of VAP was 15.1 cases per 1000 ventilator-days as reported by a systematic review and meta-analysis study in 2018 [11].

The Indonesian Health Survey 2023 reported a prevalence of 0.48% (95%CI 0.46–0.51%) for pneumonia across all ages [12]. Recent Indonesian studies in hospitalized patients with CAP revealed that the most frequently isolated pathogens were *Acinetobacter baumannii* (17.9–30.9%), *Klebsiella pneumoniae* (9.8–24.3%), and *Pseudomonas aeruginosa* (6.7–12.5%) [13,14,15,16]. The incidence of multidrug-resistant organisms in CAP patients was reported at 21.5%, with a study in Jakarta revealing a carbapenem resistance of 65% of *Acinetobacter spp.*, 30% in *Pseudomonas aeruginosa*, and 29% in *Klebsiella pneumoniae* [13,14].

A previous study reported a 65% increase in global antibiotic consumption from 2000 to 2015 [17]. The COVID-19 pandemic drove this number further, with a meta-analysis showing that 72% of COVID-19 patients received antibiotics despite bacterial co-infection being present in only 8% of cases [18]. This pattern of inappropriate use of antibiotics will contribute to the development of AMR. While empirical antibiotic therapy is required at the initial presentation of pneumonia, the ATS/IDSA Guidelines for CAP, HAP, and VAP recommend obtaining a pretreatment culture of respiratory secretions to ensure the accurate use and de-escalation of antibiotic therapy [5,6].

The prevalence of resistant bacteria varies across nations due to factors such as geographic differences and patient demographics. Understanding the distribution of bacteria and patterns of antibiotic resistance is crucial to optimizing treatment and reducing morbidity and mortality. However, studies investigating resistant bacteria among pneumonia in Indonesia remain limited and are largely concentrated in major referral centers, with minimal data available in Bandung, Indonesia. In addition, most previous studies did not comprehensively evaluate the impact of specific multidrug-resistant pathogens, particularly carbapenem-resistant *Acinetobacter baumannii* (CR-Ab), carbapenem-resistant *Klebsiella pneumoniae* (CR-Kp), difficult-to-treat resistant *Pseudomonas aeruginosa* (DTR-Psa), methicillin-resistant *Staphylococcus aureus* (MRSA) and methicillin-resistant coagulase-negative *Staphylococcus* (MRCoNS), on clinical in-hospital mortality and length of hospital stay. Therefore, this study aimed to evaluate the bacterial pathogens causing pneumonia and their antimicrobial resistance profiles among hospitalized adult patients, as well as to identify clinical factors associated with patient outcomes.

## 2. Results

### 2.1. Patients’ Characteristics

**Figure 1:**
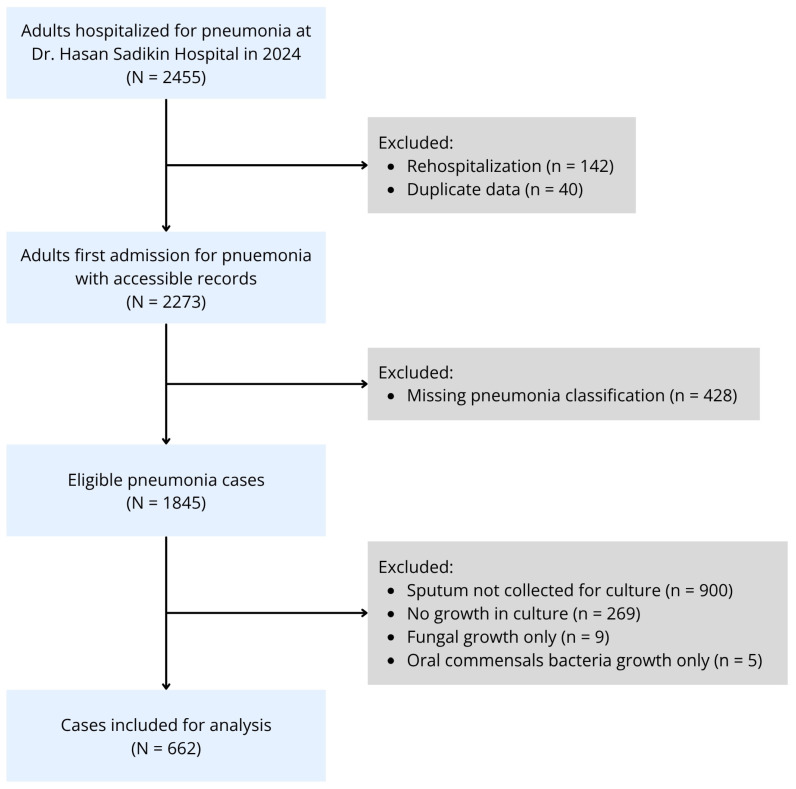
Study flow diagram.

Between January and December 2024, 2455 patients were admitted for pneumonia at Dr. Hasan Sadikin General Hospital. Of these, 662 met the eligibility criteria and were included in the final analysis (Figure 1).

The overall median age was 58 years (IQR 43–68), and 56.9% of patients were male. Community-acquired pneumonia (CAP) accounted for 63.6% (*n* = 421), followed by hospital-acquired pneumonia (HAP) at 31.7% (*n* = 210) and ventilator-associated pneumonia (VAP) at 4.7% (*n* = 31). The general characteristics across the groups are shown in Table 1.

Patients with HAP tended to be older than patients with CAP and VAP; this was also shown by the higher proportion of patients ≥60 years old in HAP, while CAP and VAP were predominated by patients <60 years old. Hypertension and diabetes mellitus were the most common comorbidities across all groups. Notably, pulmonary tuberculosis was more frequently observed in CAP than in HAP, and no cases were identified among VAP patients. Patients with VAP experienced both the longest hospital stays and the greatest proportion of in-hospital mortality.

### 2.2. Bacterial and Antimicrobial Resistance Profile

**Figure 2:**
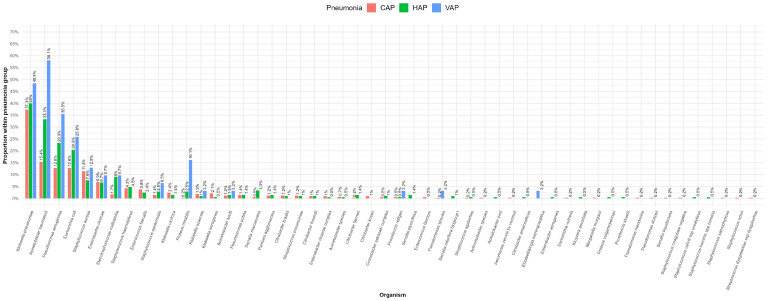
Summarized bacterial distribution in CAP, HAP, and VAP.

**Figure 3:**
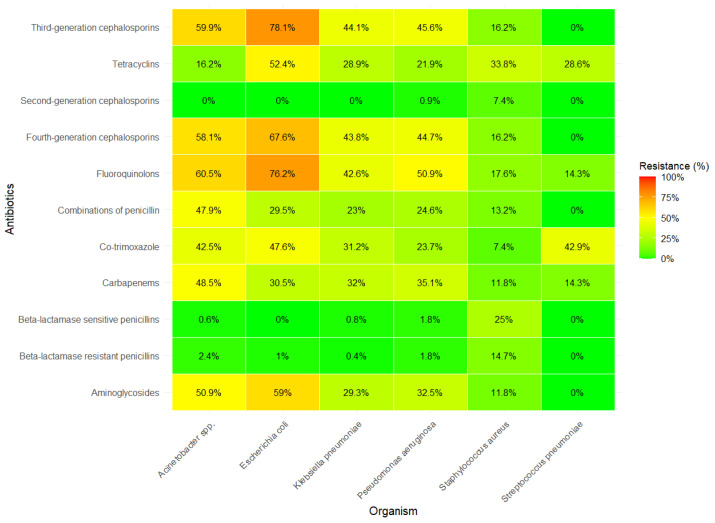
Prevalence of antimicrobial resistance for notable pathogens.

Polymicrobial growths were observed in 38.5% of the samples, yielding a total of 991 identified isolates. The distribution of bacteria across groups is shown in Figure 2 and Supplementary Material (Table S1). The most common pathogens identified were: *Klebsiella pneumoniae* (256, 38.7%), *Acinetobacter baumannii* (153, 23.1%), *Pseudomonas aeruginosa* (114, 17.2%), *Escherichia coli* (105, 15.9%), and *Staphylococcus aureus* (68, 10.3%). Substantial variability in antimicrobial resistance patterns was observed across bacterial pathogens and antibiotic classes (Figure 3). *Escherichia coli* and *Acinetobacter spp.* demonstrated the highest resistance rates overall, particularly to fluoroquinolones, third- and fourth-generation cephalosporins, and aminoglycosides, with resistance exceeding 50%. Frequent resistance was also identified in *Klebsiella pneumoniae* and *Pseudomonas aeruginosa*, especially against cephalosporins and carbapenems, whereas *Staphylococcus aureus* and *Streptococcus pneumoniae* generally exhibited lower resistance rates across most antibiotic groups.

Carbapenem-resistant *Acinetobacter baumannii* (CR-Ab) and carbapenem-resistant *Klebsiella pneumoniae* (CR-Kp) were the most frequently identified resistant pathogens, particularly in HAP (24.3% and 16.2%, respectively). CR-Ab predominated in VAP (41.9%), while CR-Kp and difficult-to-treat resistant *Pseudomonas aeruginosa* (DTR-Psa) were also common, accounting for 25.8% and 29.0%, respectively. Patients infected with resistant pathogens were generally older, a higher proportion were aged ≥60 years, and male predominance was observed across most resistance groups. Common comorbidities included hypertension and diabetes mellitus, while chronic lung disease and malignancy were also present but less consistently distributed. Methicillin-resistant *Staphylococcus aureus* (MRSA) and methicillin-resistant coagulase-negative *Staphylococcus* (MRCoNS) cases were fewer in number and showed more variable distributions across pneumonia types and comorbid conditions. Univariate analyses were conducted to explore associations between patient characteristics and specific resistant pathogens; however, multivariable analysis was not pursued, as the analysis was primarily exploratory. Detailed distributions and univariate regression analyses are provided in Supplementary Tables S2 and S3.

### 2.3. Mortality and Length of Stay

Hospital-acquired pneumonia (HAP) and ventilator-associated pneumonia (VAP) were associated with higher in-hospital mortality (Table 2) compared to community-acquired pneumonia (CAP), with adjusted risk ratios (aRR) of 1.5 and 1.8, respectively. Malignancy was also independently associated with increased mortality (aRR 1.4), while hypertension was associated with lower mortality (aRR 0.7). Age ≥ 60 years showed a trend toward higher mortality but did not reach statistical significance, and sex was not associated with the outcome. Other comorbidities, including diabetes mellitus, lung disease, and pulmonary tuberculosis, were not significantly associated with mortality after adjustment. Among resistant pathogens, no significant associations with mortality were observed for CR-Ab, CR-Kp, or DTR-Psa. Several variables, including heart disease, liver disease, kidney disease, extrapulmonary TB, MRSA, and MRCoNS, were not included in the final model. The model demonstrated moderate discrimination, with an area under the curve (AUC) of 0.6747 (95% CI 0.6324–0.7171). A sensitivity analysis using logistic regression (Supplementary Table S6) yielded similar results (AUC 0.675, 95% CI 0.632–0.717), indicating consistent model performance. Interaction analyses showed no statistically significant effect modification between pneumonia type and hypertension, malignancy, or CR-Ab infection (all *p* for interaction >0.05; Supplementary Table S7).

In the adjusted analysis (Table 3), hospital-acquired and ventilator-associated pneumonia were strongly associated with longer length of stay compared with CAP, with HAP adding 5.9 days (95% CI 3.7–8.2) and VAP adding 10.9 days (95% CI 6.0–15.8), both statistically significant. Age and gender were not significantly associated with length of stay. Among comorbidities, most showed no meaningful association, except pulmonary TB, which was associated with a shorter length of stay (−3.7 days; 95% CI −7.1 to −0.2). Regarding resistant pathogens, CR-Ab and DTR-Psa infections were significantly associated with prolonged hospitalization, increasing the length of stay by 4.3 days (95% CI 1.4–7.2) and 12.0 days (95% CI 8.0–16.1), respectively, while CR-Kp showed a non-significant trend toward longer stay. The multivariable linear regression model explained 20.1% of the variance in length of stay (R^2^ = 0.201; adjusted R^2^ = 0.189), and the mean absolute error (MAE) was 8.06 days. No statistically significant interactions were observed between pneumonia type and pulmonary tuberculosis or resistant pathogens (all *p* for interaction >0.05; Supplementary Table S10).

## 3. Discussion

This retrospective cohort study describes the microbiological characteristics, antimicrobial resistance patterns, and clinical outcomes among 662 patients with bacterial pneumonia from a tertiary hospital in Indonesia during a 1-year period. Gram-negative bacteria were the most frequently found across all pneumonia types, with resistant pathogens occurring more frequently in nosocomial pneumonia (i.e., HAP and VAP). In addition, HAP and VAP were independently associated with higher in-hospital mortality and prolonged hospitalization compared with CAP. Resistant pathogens, particularly CR-Ab and DTR-Psa, were associated primarily with longer stay rather than mortality.

The 2024 WHO Bacterial Priority Pathogens List emphasizes the increasing global threat of antibiotic-resistant bacteria by organizing clinically significant pathogens into critical, high, and medium priority groups to direct research, development, and public health efforts. Of particular concern are several Gram-negative bacteria, including *Acinetobacter baumannii*, *Pseudomonas aeruginosa*, and resistant *Enterobacterales* such as *Klebsiella pneumoniae*, which are identified as priority pathogens due to their significant impact and the scarcity of effective treatments, highlighting their crucial role in global antimicrobial resistance and pneumonia management [19]. In our cohort, Gram negative bacteria were found to be most common among isolates from patients with pneumonia. *Klebsiella pneumonia*, *Acinetobacter baumannii*, and *Pseudomonas aeruginosa* were the most prevalent, with *Klebsiella pneumonia* being the most common in both CAP and HAP, while *Acinetobacter baumannii* was the most frequent in VAP. This finding contrasts to the global estimate that identifies *Streptococcus pneumoniae* as the most prevalent cause of lower respiratory tract infections [20]. However, our findings are consistent with studies from tertiary-care settings in Asia, where Gram-negative bacteria are increasingly recognized as major causes of severe pneumonia [21,22].

Resistant Gram-negative pathogens were more frequently observed in nosocomial pneumonia (i.e., HAP and VAP). CR-Ab was the predominant resistant pathogen in both HAP and VAP, while CR-Kp and DTR-Psa were also commonly identified across the groups. Similar findings have been reported by a study from hospitals across global regions that showed that 62% of CR-Ab isolates were hospital-acquired [23]. Another study conducted in Thailand reported high carbapenem resistance rates among *Acinetobacter baumannii* (85.0% in HAP and 94.2% in VAP), *Klebsiella pneumoniae* (29.2% in HAP and 15.9% in VAP) and *Pseudomonas aeruginosa* (12.9% in HAP and 19.6% in VAP) [24].

In our study, HAP and VAP were independently associated with substantially worse clinical outcomes compared to CAP, including higher in-hospital mortality and significantly longer hospitalization. Patients with VAP experienced the highest mortality and longest hospital stay among all pneumonia types. These findings are consistent with several other studies, which demonstrated the mortality rate for nosocomial pneumonia in the range of 27.2–66.0% [25,26,27,28]. Several factors may contribute to poorer outcomes in HAP and VAP, including older age, comorbidities, ventilator use, and resistant pathogens [29,30].

In addition to their higher prevalence in nosocomial pneumonia, resistant pathogens were also associated with prolonged hospitalization. CR-Ab and DTR-Psa infections independently increased length of stay. However, despite their association with prolonged hospitalization, resistant pathogens were not independently associated with increased in-hospital mortality after adjustment. This finding suggests that resistant pathogens in this cohort may contribute more strongly to treatment complexity rather that mortality alone.

Malignancy was identified as a comorbidity associated with higher risk of in-hospital mortality. This finding is consistent with a meta-analysis of 22 studies including 3655 patients with severe pneumonia that identified a significant association between neoplasm and a higher risk of in-hospital mortality (OR 3.37, 91% CI 1.07–10.57, *p*-value 0.04) [31]. Patients with malignancy may have impaired immune responses, poorer physiological reserve, and increased susceptibility to infections due to the underlying disease or its treatment. In contrast, hypertension was associated with lower in-hospital mortality in our adjusted analysis, while chronic lung disease showed a non-significant trend toward lower mortality. Similar observations have been reported in previous studies, although the mechanisms underlying these findings remain unclear and may reflect residual confounding or differences in patient characteristics [31].

Pulmonary TB was more frequently observed among CAP patients and absent among VAP cases in our cohort. Although previous studies have reported comparable rates of concomitant pulmonary TB between CAP and nosocomial pneumonia, pulmonary TB remains an important consideration in patients presenting with community-onset respiratory infections, particularly in TB-endemic settings where it may be underrecognized [32,33,34]. The higher proportion of pulmonary TB among CAP patients in our study may therefore reflect the substantial TB burden in Indonesia and potential overlap in presentation between pulmonary TB and pneumonia. In contrast, the absence of pulmonary TB among VAP patients may reflect the distinct pathophysiology of ventilator-associated infections. Although pulmonary TB was associated with shorter hospitalization in the adjusted analysis, this finding should be interpreted cautiously due to potential unmeasured confounding, including disease severity.

A key limitation of this study is the retrospective single-center design, which may limit generalizability and introduce unmeasured confounding. Several clinically important variables, including severity-of-illness scores (e.g., CURB-65, Pneumonia Score Index), prior antibiotic exposure, timing of antimicrobial administration, and intensive care interventions, were unavailable and therefore could not be included in the analysis. Nevertheless, this study provides comprehensive data on bacterial distribution, antimicrobial resistance, and clinical outcomes across CAP, HAP, and VAP in a large cohort of hospitalized pneumonia patients from a tertiary referral center in Indonesia.

The high prevalence of antimicrobial resistance (AMR) in pneumonia is closely linked to antibiotic consumption, which creates selective pressure and promotes the emergence of resistant bacteria. Accurate and timely diagnosis is essential to ensure appropriate antibiotic treatment. To address AMR locally, antimicrobial stewardship innovations have been implemented through a digital platform system called SIMANTAP [35]. This platform strengthens efforts to raise clinicians’ awareness and enhances both the monitoring and management of antibiotic use.

## 4. Methods

### 4.1. Study Design, Settings, and Population

This retrospective cohort study was conducted at Dr. Hasan Sadikin General Hospital between January and December 2024. Dr. Hasan Sadikin General Hospital is a provincial referral hospital in West Java, a province with an estimated population of 50 million, making it the most populous in Indonesia, and 0.54% prevalence of pneumonia in 2023 [12,36]. We included adult patients (age ≥ 18 years) who were admitted with a clinical diagnosis of pneumonia during the study period. Patients were excluded if they had missing pneumonia classification, no sputum culture performed, no bacterial growth, or cultures yielding only fungal or oral flora. In cases of rehospitalization for pneumonia within the study period, only the first episode was included. The primary outcome was the characterization of bacterial pathogens identified. Secondary outcomes included length of hospital stay and in-hospital mortality.

### 4.2. Data Collection

Data from hospitalized patients with ICD-10 codes J13–J18.9 for pneumonia were extracted from electronic medical records. Demographic variables included age and sex, while clinical variables included comorbidities, length of hospital stay, and in-hospital mortality. Community-acquired pneumonia (CAP) is defined as pneumonia that develops in the community or within 48 h of hospital admission. Hospital-acquired pneumonia (HAP) occurs ≥48 h after hospitalization in patients not receiving mechanical ventilation, whereas ventilator-associated pneumonia (VAP) is a subset of HAP that develops ≥48 h after endotracheal intubation and initiation of mechanical ventilation. Comorbidities included in the analysis were chronic lung, heart, liver, and kidney disease, hypertension, diabetes mellitus, and malignancy as these are clinically relevant comorbidities recognized by the American Thoracic Society (ATS) Clinical Practice Guideline for Diagnosis and Treatment of Adults with Community-acquired Pneumonia 2019 [6]. In addition, tuberculosis (TB), both pulmonary and extrapulmonary, was also included as comorbidities due to the high TB burden in Indonesia, with an estimated incidence of 382 per 100,000 population in 2024 [37].

### 4.3. Laboratory Methods

Bacterial identification was performed on sputum samples using standard microbiological culture techniques and antibiotic susceptibility testing (AST) was conducted using the Minimum Inhibitory Concentration (MIC) method (Phoneix System, Becton Dickinson, Sparks, NV, USA) in accordance with the Clinical and Laboratory Standards Institute (CLSI) guidelines 2024 [38]. If the susceptibility interpretation was not defined by CLSI, the EUCAST guidelines were used (http://www.eucast.org/clinical_breakpoints/, accessed on 15 May 2026). Quality of microbiology procedures was monitored on a weekly basis according to the hospital standard operating procedure, manufacturer’s instructions and CLSI recommendation. Reference strains, i.e., *Escherichia coli* ATCC 25922 and *Staphylococcus aureus* ATCC 25923, were used for quality-control media performance and susceptibility testing.

In this study, AST results were categorized as either resistant or susceptible, with an intermediate result considered as resistant [39]. The list of antibiotics tested is included in the Supplementary Material (Table S10). We further classified multidrug-resistant pathogens into carbapenem-resistant pathogens (*Acinetobacter baumannii* and *Klebsiella pneumoniae*), difficult-to-treat *Pseudomonas aeruginosa* (DTR-Psa), and methicillin-resistant pathogens (coagulase-negative *Staphylococci* and *Staphylococcus aureus*). Carbapenem resistance is defined as resistance to at least one carbapenem antibiotic (ertapenem, meropenem, imipenem). DTR-Psa is defined as non-susceptibility to all of the following: piperacillin-tazobactam, ceftazidime, cefepime, aztreonam, meropenem, imipenem, ciprofloxacin, and levofloxacin. Methicillin resistance is determined phenotypically using oxacillin and/or cefoxitin as standardized surrogate agents, as methicillin itself is no longer used in routine susceptibility testing [40].

### 4.4. Data Analysis

Data analysis was performed using R statistical software (version 4.5.2; R Core Team, Vienna, Austria, 2025) and RStudio integrated development environment (version 2025.09.2; Posit, PBC, Boston, MA, USA). Descriptive statistics was used to summarize characteristics, with categorical variables presented as frequencies and percentages and continuous variables as medians and interquartile ranges (IQR). Bacterial distribution and antimicrobial resistance patterns were analyzed at the isolate level, using the total number of isolates as the denominator. Only one isolate per species was included for each patient. Clinical outcome analyses were conducted at the patient level. In polymicrobial infections, patients could belong to multiple MDR organism groups, which were included as separate binary variables within the same patient. Differences between characteristics were tested for statistical significance using Pearson’s chi-square or Fisher’s exact test for categorical variables and Wilcoxon rank-sum test for continuous variables.

Multivariable logistic regression was used to identify factors associated with in-hospital mortality, while linear regression analysis was used to identify predictors of length of hospital stay. The multivariable model was constructed using purposeful selection, including variables significant in bivariate analysis and those deemed clinically relevant based on the prior literature. Adjusted odds ratios (ORs) and beta coefficients with 95% confidence intervals (CIs) and *p*-values were reported. Statistical significance was defined as a *p*-value < 0.05. Given the relatively high incidence of in-hospital mortality, modified Poisson regression with robust standard errors was additionally performed to estimate adjusted risk ratios (RR) as a sensitivity analysis. Model discrimination for mortality was assessed using receiver operating characteristic (ROC) curves and the area under the curve (AUC). In addition, potential effect modification was assessed by including interaction terms between key variables in the multivariable models.

## 5. Conclusions

In this cohort of hospitalized adults with bacterial pneumonia at a tertiary hospital in West Java, Indonesia, Gram-negative bacteria were the most frequently found across all pneumonia types, with resistant pathogens occurring more frequently in nosocomial pneumonia (i.e., HAP and VAP). HAP and VAP were independently associated with higher in-hospital mortality and prolonged hospitalization compared to CAP. In addition, CR-Ab and DTR-Psa infections were associated with a longer length of stay, highlighting the substantial healthcare burden posed by resistant pathogens. These findings underscore the importance of continuous local microbiological surveillance, antimicrobial stewardship, and infection prevention strategies to guide empirical antimicrobial therapy and optimize clinical outcomes among hospitalized pneumonia patients.

## Figures and Tables

**Table 1:** Baseline characteristics of the study population.

	CAP (*N* = 421) *n* (%)	HAP (*N* = 210) *n* (%)	VAP (*N* = 31) *n* (%)
Age (median, IQR)	57 (43–67)	60 (46–69)	53 (38–62)
Age ≥ 60 years	176 (41.8)	110 (52.4)	9 (29.0)
Male	230 (54.6)	131 (62.4)	16 (51.6)
Comorbidities			
Hypertension	190 (45.1)	93 (44.3)	13 (41.9)
Diabetes mellitus	83 (19.7)	34 (16.2)	4 (12.9)
Chronic kidney disease	56 (13.3)	28 (13.3)	4 (12.9)
Chronic lung disease	71 (16.9)	9 (4.3)	1 (3.2)
Chronic heart disease	38 (9.0)	34 (16.2)	4 (12.9)
Pulmonary tuberculosis	53 (12.6)	6 (2.9)	0 (0)
Malignancy	76 (18.1)	53 (25.2)	3 (9.7)
Extrapulmonary tuberculosis	33 (7.8)	5 (2.4)	0 (0)
Chronic liver disease	8 (1.9)	6 (2.9)	0 (0)
Length of hospital stay, days (median, IQR)	8 (5–12)	15 (10–26)	19 (14–40)
In-hospital mortality	125 (29.7)	101 (48.1)	18 (58.1)

CAP, community-acquired pneumonia; HAP, hospital-acquired pneumonia; IQR, interquartile range; VAP, ventilator-associated pneumonia.

**Table 2:** Factors associated with in-hospital mortality.

	Died (*N* = 244) n (%)	Survived (*N* = 418) n (%)	aRR (95% CI)	*p*-Value
Pneumonia type				
CAP	125 (29.7)	296 (70.3)	Ref	
HAP	101 (48.1)	109 (51.9)	1.5 (1.1–1.9)	**0.009**
VAP	18 (58.1)	13 (41.9)	1.8 (1.0–3.0)	**0.029**
Age ≥ 60	117 (39.7)	178 (60.3)	1.3 (1.0–1.6)	0.091
Male	134 (35.5)	243 (64.5)	0.9 (0.7–1.1)	0.285
Comorbidities				
Hypertension	90 (30.4)	206 (69.6)	0.7 (0.5–0.9)	**0.020**
Diabetes mellitus	45 (37.2)	76 (62.8)	1.2 (0.8–1.6)	0.398
Chronic lung disease	17 (21.0)	64 (79.0)	0.6 (0.4–1.0)	0.092
Chronic heart disease	28 (36.8)	48 (63.2)	–	–
Chronic liver disease	6 (42.9)	8 (57.1)	–	–
Chronic kidney disease	32 (36.4)	56 (63.6)	–	–
Malignancy	66 (50.0)	66 (50.0)	1.4 (1.1–1.9)	**0.020**
Pulmonary TB	22 (37.3)	37 (62.7)	1.2 (0.7–1.8)	0.490
Extrapulmonary TB	16 (42.1)	22 (57.9)	–	–
Resistant pathogens				
CR-Ab	45 (48.4)	48 (51.6)	1.2 (0.8–1.7)	0.291
CR-Kp	34 (42.5)	46 (57.5)	1.0 (0.7–1.5)	0.902
DTR-Psa	21 (47.7)	23 (52.3)	1.0 (0.6–1.6)	0.979
MRSA	5 (50.0)	5 (50.0)	–	–
MRCoNS	12 (29.3)	29 (70.7)	–	–

CAP, community-acquired pneumonia; CR-Ab, carbapenem-resistant *Acinetobacter baumannii*; CR-Kp, carbapenem-resistant *Klebsiella pneumoniae*; DTR-Psa, difficult-to-treat *Pseudomonas aeruginosa*; HAP, hospital-acquired pneumonia; MRCoNS, methicillin-resistant coagulase-negative *Staphylococcus*; MRSA, methicillin-resistant *Staphylococcus aureus*; VAP, ventilator-associated pneumonia. ‘–’ indicates variable not included in the final model; ‘Ref’ indicates the reference category. Significant *p*-values (<0.05) are shown in bold.

**Table 3:** Factors associated with length of stay.

	Length of Stay, Days (Median, IQR)	Adjusted Beta (95% CI)	*p*-Value
Pneumonia type			
CAP	8 (5–12)		
HAP	15 (10–26)	5.9 (3.7–8.2)	**<0.001**
VAP	19 (14–41)	10.9 (6.0–15.8)	**<0.001**
Age			
<60	9 (6–17)	Ref	
≥60	10 (6–20)	−0.2 (−2.2–1.8)	0.833
Gender			
Male	10 (6–17)	−1.1 (−3.1–0.8)	0.256
Female	10 (6–21)	Ref	
Comorbidities			
Hypertension	10 (7–20)	–	–
Diabetes mellitus	10 (6–20)	0.9 (−1.6–3.4)	0.489
Chronic lung disease	7 (5–12)	−2.2 (−5.2–0.8)	0.256
Chronic heart disease	10 (7–17)	–	–
Chronic liver disease	9 (7–13)	–	–
Chronic kidney disease	9 (6–17)	–	–
Malignancy	10 (6–17)	–	–
Pulmonary TB	6 (5–11)	−3.7 (−7.1–(−0.2))	**0.036**
Extrapulmonary TB	9 (5–20)	–	–
Resistant pathogens			
CR-Ab	20 (10–28)	4.3 (1.4–7.2)	**0.004**
CR-Kp	12 (6–27)	2.6 (−0.4–5.5)	0.095
DTR-Psa	24 (12–45)	12.0 (8.0–16.1)	**<0.001**
MRSA	10 (5–13)	–	–
MRCoNS	10 (7–15)	–	–

CAP, community-acquired pneumonia; CR-Ab, carbapenem-resistant *Acinetobacter baumannii*; CR-Kp, carbapenem-resistant *Klebsiella pneumoniae*; DTR-Psa, difficult-to-treat *Pseudomonas aeruginosa*; HAP, hospital-acquired pneumonia; MRCoNS, methicillin-resistant coagulase-negative *Staphylococcus*; MRSA, methicillin-resistant *Staphylococcus aureus*; VAP, ventilator-associated pneumonia. ‘–’ indicates variable not included in the final model; β coefficient indicates change in length of stay (days); ‘Ref’ indicates the reference category. Significant *p*-values (<0.05) are shown in bold.

## Data Availability

Dataset available on request from the authors.

## Supplementary Materials

The following supporting information can be downloaded at: https://www.mdpi.com/article/10.3390/antibiotics15060582/s1, Table S1. Bacterial distribution. Table S2. Characteristics of patients with specific resistant pathogen infections. Table S3. Characteristics of patients with specific resistant pathogen infections. Table S4. In-hospital mortality. Table S5. Predictors for in-hospital mortality. Table S6. Sensitivity analysis with multivariable logistic regression. Table S7. Multivariable model with interaction terms for in-hospital mortality. Table S8. Length of stay. Table S9. Predictors of length of hospital stay. Table S10. Multivariable model with interaction terms for hospital length of stay. Table S11. List of antibiotics tested.

## Abbreviations

The following abbreviations are used in this manuscript:

CAP	Community-acquired pneumonia
HAP	Hospital-acquired pneumonia
VAP	Ventilator-associated pneumonia
CR-Ab	Carbapenem-resistant *Acinetobacter baumannii*
CR-Kp	Carbapenem-resistant *Klebsiella pneumoniae*
DTR-Psa	Difficult-to-treat *Pseudomonas aeruginosa*
AMR	Antimicrobial resistance
WHO	World Health Organization
COVID-19	Coronavirus disease 2019
ATS	American Thoracic Society
IDSA	Infectious Disease Society of America
MDR	Multidrug-resistant
ICD	International classification of disease
TB	Tuberculosis
AST	Antibiotic susceptibility testing
CLSI	Clinical and Laboratory Standards Institute
OR	Odds ratio
CI	Confidence interval
MRCoNS	Methicillin-resistant coagulase-negative *Staphylococcus*
MRSA	Methicillin-resistant Staphylococcus aureus
IQR	Interquartile range
