# Supplementary material for: Antimicrobial Resistance and Clinical Outcome Among Hospitalized Bacterial Pneumonia: A Retrospective Cohort Study in Indonesian Tertiary Hospital
Source: Antibiotics (Basel). 2026 Jun 8;15(6):582. doi: 10.3390/antibiotics15060582 (PMC13296290; doi:10.3390/antibiotics15060582)
Supplement: Supplementary file 1 [file antibiotics-15-00582-s001.zip › antibiotics-4233656-supplementary.pdf]

Table S1. Bacterial distribution.

	CAP (N = 545) n (%)	HAP (N = 372) n (%)	VAP (N = 74) n (%)
**Gram negative bacteria**			
Klebsiella pneumoniae	157 (28.8)	84 (22.6)	15 (20.3)
Acinetobacter baumannii	65 (11.9)	70 (18.8)	18 (24.3)
Pseudomonas aeruginosa	54 (9.9)	49 (13.2)	11 (14.9)
Escherichia coli	54 (9.9)	43 (11.6)	8 (10.8)
Enterobacter cloacae	29 (5.3)	14 (3.8)	3 (4.1)
Stenotophomonas maltophilia	7 (1.3)	19 (5.1)	3 (4.1)
Klebsiella oxytoca	10 (1.8)	3 (0.8)	0 (0)
Proteus mirabilis	1 (0.2)	6 (1.6)	5 (6.8)
Klebsiella ozaenae	8 (1.5)	2 (0.5)	1 (1.4)
Klebsiella aerogenes	9 (1.7)	1 (0.3)	0 (0)
Pseudomonas putida	6 (1.1)	3 (0.8)	0 (0)
Acinetobacter lwofii	5 (0.9)	3 (0.8)	1 (1.4)
Serratia marcescens	2 (0.4)	7 (1.9)	0 (0)
Pantoea agglomerans	5 (0.9)	3 (0.8)	0 (0)
Citrobacter braakii	5 (0.9)	2 (0.5)	0 (0)
Citrobacter freundii	4 (0.7)	2 (0.5)	0 (0)
Enterobacter cloacae complex	4 (0.7)	1 (0.3)	0 (0)
Providencia rettgeri	2 (0.4)	1 (0.3)	1 (1.4)
Acinetobacter sp	3 (0.6)	1 (0.3)	0 (0)
Citrobacter koseri	4 (0.7)	0 (0)	0 (0)
Cronobacter sakazaki complex	2 (0.4)	2 (0.5)	0 (0)
Citrobacter farmeri	1 (0.2)	3 (0.8)	0 (0)
Serratia plymuthica	0 (0)	3 (0.8)	0 (0)
Serratia odorifera biogroup 1	0 (0)	2 (0.5)	0 (0)
Pseudomonas sp	1 (0.2)		1 (1.4)
Acinetobacter junii	0 (0)	1 (0.3)	0 (0)
Pseudomonas mendocina	1 (0.2)	0 (0)	0 (0)
Morganella morganii	1 (0.2)	0 (0)	0 (0)
Serratia liquefaciens	1 (0.2)	0 (0)	0 (0)
Escherichia vulneris	1 (0.2)	0 (0)	0 (0)
Providencia stuartii	0 (0)	1 (0.3)	0 (0)
Elizabethkingia meningoseptica	0 (0)	0 (0)	1 (1.4)
Enterobacter aerogenes	0 (0)	1 (0.3)	0 (0)
Kluyvera ascorbata	0 (0)	1 (0.3)	0 (0)
Aeromonas veronii	1 (0.2)	0 (0)	0 (0)
Proteus vulgaris/penneri	0 (0)	1 (0.3)	0 (0)
Achromobacter sp	1 (0.2)	0 (0)	0 (0)
Pseudomonas stutzeri	1 (0.2)	0 (0)	0 (0)
Citrobacter amalonaticus	0 (0)	1 (0.3)	0 (0)
**Gram positive bacteria**			
Staphylococcus aureus	48 (8.8)	16 (4.3)	4 (5.4)
Staphylococcus haemolyiticus	18 (3.3)	10 (2.7)	0 (0)
Enterococcus faecalis	16 (2.9)	5 (1.3)	0 (0)
Staphylococcus epidermidis	6 (1.1)	6 (1.6)	2 (2.7)
Streptococcus pneumoniae	5 (0.9)	2 (0.5)	0 (0)
Enterococcus faecium	2 (0.4)	0 (0)	0 (0)
Streptococcus agalactiae	1 (0.2)	1 (0.3)	0 (0)
Staphylococcus coagulase negative	1 (0.2)	0 (0)	0 (0)
Staphylococcus hominis spp hominis	0 (0)	1 (0.3)	0 (0)
Streptococcus dysgalactiae	1 (0.2)	0 (0)	0 (0)
Staphylococcus sciuri	1 (0.2)	0 (0)	0 (0)
Staphylococcus saprophyticus	1 (0.2)	0 (0)	0 (0)
Staphylococcus cohnii spp urealyticus	0 (0)	1 (0.3)	0 (0)

Table S2. Characteristics of patients with specific resistant pathogen infections.

	CR-Ab (N=93)		CR-Kp (N=80)		DTR-Psa (N=44)		MRCoNS (N=41)		MRSA (N=10)	
	**n (%)**	**p-value**	**n (%)**	**p-value**	**n (%)**	**p-value**	**n (%)**	**p-value**	**n (%)**	**p-value**
**Pneumonia type**		**<0.001**		**0.002**		**<0.001**		0.599		0.408
CAP (N=421)	29 (6.9)		38 (9.0)		11 (2.6)		24 (5.7)		7 (1.7)	
HAP (N=210)	51 (24.3)		34 (16.2)		24 (11.4)		16 (7.6)		2 (1.0)	
VAP (N=31)	13 (41.9)		8 (25.8)		9 (29.0)		1 (3.2)		1 (3.2)	
**Age, years (median, IQR)**	60 (48 - 69)	0.115	63 (50 - 70)	**0.028**	56 (41 - 68)	0.593	61 (53 - 71)	0.126	55 (51 - 66)	0.942
**Age ≥60 (N=295)**	48 (16.3)	0.140	46 (15.6)	**0.013**	19 (6.4)	0.849	23 (7.8)	0.125	4 (1.4)	1.000
**Male (N=377)**	55 (14.6)	0.645	50 (13.3)	0.285	27 (7.2)	0.540	28 (7.4)	0.130	3 (0.8)	0.109
**Comorbidities**										
Hypertension (N=296)	47 (15.9)	0.228	38 (12.8)	0.602	16 (5.4)	0.245	22 (7.4)	0.238	4 (1.4)	1.000
Diabetes mellitus (N=121)	16 (13.2)	0.767		0.301	9 (7.4)	0.703	6 (5.0)	0.530	3 (2.5)	0.401
Chronic lung disease (N=81)	6 (7.4)	0.066	18 (14.9)	0.774	2 (2.5)	0.107	8 (9.9)	0.142	0 (0)	0.620
Chronic heart disease (N=76)	10 (13.2)	0.812	9 (11.1)	0.760	2 (2.6)	0.135	6 (7.9)	0.454	1 (1.3)	1.000
Chronic liver disease (N=14)	1 (7.1)	0.705	10 (13.2)	0.682	0 (0)	0.615	2 (14.3)	0.213	1 (7.1)	0.194
Chronic kidney disease (N=88)	16 (18.2)	0.231	2 (14.3)	0.632	7 (8.0)	0.597	6 (6.8)	0.794	2 (2.3)	0.630
Malignancy (N=132)	13 (9.8)	0.121	12 (13.6)	0.988	9 (6.8)	0.929	7 (5.3)	0.635	3 (2.3)	0.426
Pulmonary TB (N=59)	4 (6.8)	0.092	16 (12.1)	0.716	2 (3.4)	0.415	3 (5.1)	1.000	0 (0)	1.000
Extrapulmonary TB (N=38)	4 (10.5)	0.520	8 (13.6)	0.072	1 (2.6)	0.503	0 (0)	0.160	0 (0)	1.000

CAP, community-acquired pneumonia; CR-Ab, carbapenem-resistant *Acinetobacter baumannii*; CR-Kp, carbapenem-resistant *Klebsiella pneumoniae*; DTR-Psa, difficult-to-treat *Pseudomonas aeruginosa*; HAP, hospital-acquired pneumonia; MRCoNS, methicillin-resistant coagulase negative *Staphylococcus*; MRSA, methicillin-resistant *Staphylococcus aureus*; VAP, ventilator-associated pneumonia.

Table S3. Characteristics of patients with specific resistant pathogen infections.

	CR-Ab (N=93)		CR-Kp (N=80)		DTR-Psa (N=44)		MRCoNS (N=41)		MRSA (N=10)	
	cRR (95% CI)	p-value	cRR (95% CI)	p-value	cRR (95% CI)	p-value	cRR (95% CI)	p-value	cRR (95% CI)	p-value
**Pneumonia type**										
CAP	Ref		Ref		Ref		Ref		Ref	
HAP	1.2 (1.1 - 1.3)	**<0.001**	1.8 (1.1 - 2.8)	**0.013**	4.4 (2.1 - 8.9)	**<0.001**	1.3 (0.7 - 2.5)	0.369	0.6 (0.1 - 2.8)	0.487
VAP	1.4 (1.3 - 1.6)	**<0.001**	3.0 (1.4 - 6.3)	**0.005**	11.5 (4.8 - 27.7)	**<0.001**	0.6 (0.1 - 4.3)	0.599	2.0 (0.2 - 16.3)	0.515
**Age ≥60**	1.3 (0.9 - 2.0)	0.177	1.7 (1.1 - 2.6)	**0.022**	0.9 (0.5 - 1.7)	0.847	1.6 (0.9 - 2.9)	0.143	0.8 (0.2 - 2.9)	0.769
**Male**	1.1 (0.7 - 1.7)	0.661	1.3 (0.8 - 2.0)	0.312	1.2 (0.7 - 2.2)	0.549	1.6 (0.8 - 3.2)	0.144	0.3 (0.1 - 1.3)	0.103
**Comorbidities**										
Hypertension	1.3 (0.8 - 1.9)	0.265	1.1 (0.7 - 1.70	0.625	0.7 (0.4 - 1.3)	0.264	1.4 (0.8 - 2.6)	0.255	0.8 (0.2 - 2.9)	0.761
Diabetes mellitus	0.9 (0.5 - 1.6)	0.784	1.3 (0.8 - 2.2)	0.333	1.1 (0.6 - 2.4)	0.713	0.8 (0.3 - 1.8)	0.544	1.9 (0.5 - 7.4)	0.347
Chronic lung disease	0.5 (0.2 - 1.1)	0.095	0.9 (0.5 - 1.8)	0.784	0.3 (0.1 - 1.4)	0.137	1.7 (0.8 - 3.8)	0.162	NA	NA
Chronic heart disease	0.9 (0.5 - 1.8)	0.822	1.1 (0.6 - 2.1)	0.779	0.4 (0.1 - 1.5)	0.166	1.3 (0.6 - 3.1)	0.53	0.9 (1.0 - 6.7)	0.882
Chronic liver disease	0.5 (0.1 - 3.6)	0.493	1.2 (0.3 - 4.8)	0.813	NA	NA	2.4 (0.6 - 9.8)	0.234	5.1 (0.7 - 40.5)	0.121
Chronic kidney disease	1.4 (0.8 - 2.3)	0.271	1.1 (0.6 - 2.1)	0.657	1.2 (0.5 - 2.8)	0.613	1.1 (0.5 - 2.7)	0.803	1.6 (0.3 - 7.7)	0.538
Malignancy	0.7 (0.4 - 1.2)	0.152	1.0 (0.6 - 1.7)	0.995	1.0 (0.5 - 2.1)	0.936	0.8 (0.4 - 1.9)	0.643	1.7 (0.4 - 6.6)	0.433
Pulmonary TB	0.5 (0.2 - 1.2)	0.127	1.1 (0.5 - 2.4)	0.736	0.5 (0.1 - 2.0	0.319	0.8 (0.2 - 2.6)	0.718	NA	NA
Extrapulmonary TB	0.7 (0.3 - 2.0)	0.55	0.2 (0.0 - 1.5)	0.118	0.4 (0.1 - 2.8)	0.34	NA	NA	NA	NA

CAP, community-acquired pneumonia; CR-Ab, carbapenem-resistant *Acinetobacter baumannii*; CR-Kp, carbapenem-resistant *Klebsiella pneumoniae*; DTR-Psa, difficult-to-treat *Pseudomonas aeruginosa*; HAP, hospital-acquired pneumonia; MRCoNS, methicillin-resistant coagulase negative *Staphylococcus*; MRSA, methicillin-resistant *Staphylococcus aureus*; VAP, ventilator-associated pneumonia.

'NA' indicates estimates could not be computed due to zero events in one group.

Table S4. In-hospital mortality.

	Died (N=244)<br>n (%)	Survived (N=418)<br>n (%)	p-value
**Pneumonia type**			**<0.001**
CAP	125 (29.7)	296 (70.3)	
HAP	101 (48.1)	109 (51.9)	
VAP	18 (58.1)	13 (41.9)	
**Age, years (median, IQR)**	59 (47 - 69)	56 (41 - 67)	**0.035**
**Age ≥60**	117 (39.7)	178 (60.3)	0.18
**Male**	134 (35.5)	243 (64.5)	0.42
**Comorbidities**			
Hypertension	90 (30.4)	206 (69.6)	**0.002**
Diabetes mellitus	45 (37.2)	76 (62.8)	0.914
Chronic lung disease	17 (21.0)	64 (79.0)	**0.002**
Chronic heart disease	28 (36.8)	48 (63.2)	0.998
Chronic liver disease	6 (42.9)	8 (57.1)	0.638
Chronic kidney disease	32 (36.4)	56 (63.6)	0.918
Malignancy	66 (50.0)	66 (50.0)	**<0.001**
Pulmonary TB	22 (37.3)	37 (62.7)	0.943
Extrapulmonary TB	16 (42.1)	22 (57.9)	0.49
**Resistant pathogens**			
CR-Ab	45 (48.4)	48 (51.6)	**0.013**
CR-Kp	34 (42.5)	46 (57.5)	0.265
DTR-Psa	21 (47.7)	23 (52.3)	0.122
MRSA	5 (50.0)	5 (50.0)	0.511
MRCoNS	12 (29.3)	29 (70.7)	0.298

CAP, community-acquired pneumonia; CR-Ab, carbapenem-resistant *Acinetobacter baumannii*; CR-Kp, carbapenem-resistant *Klebsiella pneumoniae*; DTR-Psa, difficult-to-treat *Pseudomonas aeruginosa*; HAP, hospital-acquired pneumonia; MRCoNS, methicillin-resistant coagulase negative *Staphylococcus*; MRSA, methicillin-resistant *Staphylococcus aureus*; VAP, ventilator-associated pneumonia.

Table S5. Predictors for in-hospital mortality

	cRR (95% CI)	p-value	aRR (95% CI)	p-value
**Pneumonia type**				
CAP	Ref		Ref	
HAP	1.6 (1.2 – 2.1)	**<0.001**	1.5 (1.1 – 1.9)	**0.009**
VAP	1.9 (1.1 – 3.2)	**0.012**	1.8 (1.0 – 3.0)	**0.029**
**Age ≥60**	1.2 (0.9 – 1.5)	0.270	1.3 (1.0 – 1.6)	0.091
**Male**	0.9 (0.7 – 1.2)	0.498	0.9 (0.7 – 1.1)	0.285
**Comorbidities**				
Hypertension	0.7 (0.6 – 0.9)	**0.016**	0.7 (0.5 – 0.9)	**0.020**
Diabetes mellitus	1.0 (0.7 – 1.4)	0.932	1.2 (0.8 – 1.6)	0.398
Chronic lung disease	0.5 (0.3 – 0.9)	**0.014**	0.6 (0.4 – 1.0)	0.092
Chronic heart disease	1.0 (0.7 – 1.5)	0.990	–	–
Chronic liver disease	1.2 (0.5 – 2.6)	0.704	–	–
Chronic kidney disease	1.0 (0.7 – 1.4)	0.947	–	–
Malignancy	1.5 (1.1 – 2.0)	**0.005**	1.4 (1.1 – 1.9)	**0.020**
Pulmonary TB	1.0 (0.7 – 1.6)	0.944	1.2 (0.7 – 1.8)	0.490
Extrapulmonary TB	1.2 (0.7 – 1.9)	0.576	–	–
**Resistant pathogens**				
CR-Ab	1.4 (1.0 – 1.9)	**0.047**	1.2 (0.8 – 1.7)	0.291
CR-Kp	1.2 (0.8 – 1.7)	0.367	1.0 (0.7 – 1.5)	0.902
DTR-Psa	1.3 (0.8 – 2.1)	0.216	1.0 (0.6 – 1.6)	0.979
MRSA	1.4 (0.6 – 3.3)	0.488	–	–
MRCoNS	0.8 (0.4 – 1.4)	0.415	–	–

CAP, community-acquired pneumonia; CR-Ab, carbapenem-resistant *Acinetobacter baumannii*; CR-Kp, carbapenem-resistant *Klebsiella pneumoniae*; DTR-Psa, difficult-to-treat *Pseudomonas aeruginosa*; HAP, hospital-acquired pneumonia; MRCoNS, methicillin-resistant coagulase negative *Staphylococcus*; MRSA, methicillin-resistant *Staphylococcus aureus*; VAP, ventilator-associated pneumonia.
'–' indicates variable not included in the final model; 'ref' indicates the reference category.

Table S6. Sensitivity analysis with multivariable logistic regression

	aRR (95% CI)	p-value
**Pneumonia type**		
CAP	Ref	
HAP	1.9 (1.3 - 2.8)	**<0.001**
VAP	3.0 (1.3 – 6.8)	**0.008**
**Age ≥60**	1.5 (1.0 – 2.1)	**0.029**
**Male**	0.8 (0.6 – 1.1)	0.181
**Comorbidities**		
Hypertension	0.6 (0.4 – 0.8)	**0.003**
Diabetes mellitus	1.3 (0.8 – 1.9)	0.310
Chronic lung disease	0.5 (0.3 – 0.9)	**0.038**
Chronic heart disease	–	–
Chronic liver disease	–	–
Chronic kidney disease	–	–
Malignancy	1.9 (1.3 – 2.9)	**0.002**
Pulmonary TB	1.3 (0.7 – 2.3)	0.388
Extrapulmonary TB	–	–
**Resistant pathogens**		
CR-Ab	1.4 (0.9 – 2.3)	0.171
CR-Kp	1.1 (0.6 – 1.8)	0.809
DTR-Psa	1.0 (0.5 – 2.0)	1.000
MRSA	–	–
MRCoNS	–	–

CAP, community-acquired pneumonia; CR-Ab, carbapenem-resistant *Acinetobacter baumannii*; CR-Kp, carbapenem-resistant *Klebsiella pneumoniae*; DTR-Psa, difficult-to-treat *Pseudomonas aeruginosa*; HAP, hospital-acquired pneumonia; MRCoNS, methicillin-resistant coagulase negative *Staphylococcus*; MRSA, methicillin-resistant *Staphylococcus aureus*; VAP, ventilator-associated pneumonia.
'–' indicates variable not included in the final model; 'ref' indicates the reference category.

Table S7. Multivariable model with interaction terms for in-hospital mortality

	aRR (95% CI)	p-value
**Pneumonia type**		
CAP	Ref	
HAP	1.6 (1.0 – 2.4)	**0.040**
VAP	2.2 (0.9 – 4.6)	0.052
**Age ≥60**	1.3 (1.0 – 1.7)	0.076
**Male**	0.9 (0.7 – 1.1)	0.306
**Comorbidities**		
Hypertension	0.7 (0.5 – 1.0)	**0.041**
Diabetes mellitus	1.1 (0.8 – 1.6)	0.455
Chronic lung disease	0.6 (0.4 – 1.0)	0.085
Chronic heart disease	–	–
Chronic liver disease	–	–
Chronic kidney disease	–	–
Malignancy	1.6 (1.1 – 2.4)	**0.016**
Pulmonary TB	1.2 (0.7 – 1.8)	0.467
Extrapulmonary TB	–	–
**Resistant pathogens**		
CR-Ab	1.5 (0.8 – 2.6)	0.189
CR-Kp	1.1 (0.7 – 1.5)	0.772
DTR-Psa	1.0 (0.6 – 1.6)	0.974
MRSA	–	–
MRCoNS	–	–
**Interaction**		
**Pneumonia * Hypertension**		
HAP	1.1 (0.6 – 1.9)	0.803
VAP	1.9 (0.6 – 5.4)	0.243
**Pneumonia * Malignancy**		
HAP	0.7 (0.4 – 1.3)	0.307
VAP	0.6 (0.1 – 2.6)	0.560
**Pneumonia * CR-Ab**		
HAP	0.8 (0.4 – 1.8)	0.620
VAP	0.3 (0.1 – 1.1)	0.072

CAP, community-acquired pneumonia; CR-Ab, carbapenem-resistant *Acinetobacter baumannii*; CR-Kp, carbapenem-resistant *Klebsiella pneumoniae*; DTR-Psa, difficult-to-treat *Pseudomonas aeruginosa*; HAP, hospital-acquired pneumonia; MRCoNS, methicillin-resistant coagulase negative *Staphylococcus*; MRSA, methicillin-resistant *Staphylococcus aureus*; VAP, ventilator-associated pneumonia.
'–' indicates variable not included in the final model; 'ref' indicates the reference category; ; '*' are interaction terms.

Table S8. Length of stay.

	Length of stay, days (median, IQR)	p-value
**Pneumonia type**		**<0.001**
CAP	8 (5 – 12)	
HAP	15 (10 – 26)	
VAP	19 (14 – 41)	
**Age**		0.268
<60	9 (6 – 17)	
≥60	10 (6 – 20)	
**Gender**		0.294
Male	10 (6 – 17)	
Female	10 (6 – 21)	
**Comorbidities**		
Hypertension	10 (7 – 20)	0.138
Diabetes mellitus	10 (6 – 20)	0.908
Chronic lung disease	7 (5 – 12)	**0.001**
Chronic heart disease	10 (7 – 17)	0.853
Chronic liver disease	9 (7 – 13)	0.702
Chronic kidney disease	9 (6 – 17)	0.975
Malignancy	10 (6 – 17)	0.512
Pulmonary TB	6 (5 – 11)	**<0.001**
Extrapulmonary TB	9 (5 – 20)	0.531
**Resistant pathogens**		
CR-An	20 (10 – 28)	**<0.001**
CR-Kp	12 (6 – 27)	**0.033**
DTR-Psa	24 (12 – 45)	**<0.001**
MRSA	10 (5 – 13)	0.619
MRCoNS	10 (7 – 15)	0.808

CAP, community-acquired pneumonia; CR-Ab, carbapenem-resistant *Acinetobacter baumannii*; CR-Kp, carbapenem-resistant *Klebsiella pneumoniae*; DTR-Psa, difficult-to-treat *Pseudomonas aeruginosa*; HAP, hospital-acquired pneumonia; MRCoNS, methicillin-resistant coagulase negative *Staphylococcus*; MRSA, methicillin-resistant *Staphylococcus aureus*; VAP, ventilator-associated pneumonia.

Table S9. Predictors of length of hospital stay

	β coefficient	p-value	Adjusted β coefficient	p-value
**Pneumonia type**				
CAP	Ref		Ref	
HAP	8.4 (6.3 – 10.6)	**<0.001**	5.9 (3.7 – 8.2)	**<0.001**
VAP	17.0 (12.1 – 21.8)	**<0.001**	10.9 (6.0 – 15.8)	**<0.001**
**Age**				
<60	Ref		Ref	
≥60	0.4 (-1.7 - 2.5)	0.706	-0.2 (-2.2 - 1.8)	0.833
**Gender**				
Male	-0.8 (-2.9 - 1.4)	0.478	-1.1 (-3.1 - 0.8)	0.256
Female	Ref		Ref	
**Comorbidities**				
Hypertension	2.1 (-0.1 - 4.2)	0.059	–	–
Diabetes mellitus	0.9 (-1.8 - 3.6)	0.520	0.9 (-1.6 - 3.4)	0.489
Chronic lung disease	-5.2 (-8.4 - (-1.9))	**0.002**	-2.2 (-5.2 - 0.8)	0.256
Chronic heart disease	-0.9 (-4.2 - 2.4)	0.592	–	–
Chronic liver disease	-2.7 (-10.1 - 4.6)	0.468	–	–
Chronic kidney disease	1.2 (-1.9 - 4.3)	0.455	–	–
Malignancy	-0.8 (-3.5 - 1.8)	0.542	–	–
Pulmonary TB	-6.5 (-10.2 - (-2.8))	**<0.001**	-3.7 (-7.1 - (-0.2)	**0.036**
Extrapulmonary TB	-1.2 (-5.8 - 3.3)	0.596	–	–
**Resistant pathogens**				
CR-Ab	9.2 (6.2 - 12.1)	**<0.001**	4.3 (1.4 - 7.2)	**0.004**
CR-Kp	5.5 (2.3 - 8.8)	**<0.001**	2.6 (-0.4 - 5.5)	0.095
DTR-Psa	17.3 (13.2 - 21.3)	**<0.001**	12.0 (8.0 - 16.1)	**<0.001**
MRSA	0.5 (-8.2 - 9.1)	0.917	–	–
MRCoNS	0.1 (-4.3 - 4.5)	0.957	–	–

CAP, community-acquired pneumonia; CR-Ab, carbapenem-resistant *Acinetobacter baumannii*; CR-Kp, carbapenem-resistant *Klebsiella pneumoniae*; DTR-Psa, difficult-to-treat *Pseudomonas aeruginosa*; HAP, hospital-acquired pneumonia; MRCoNS, methicillin-resistant coagulase negative *Staphylococcus*; MRSA, methicillin-resistant *Staphylococcus aureus*; VAP, ventilator-associated pneumonia.
'–' indicates variable not included in the final model; β coefficient indicates change in length of stay (days); 'ref' indicates the reference category.

Table S10. Multivariable model with interaction terms for hospital length of stay

	Adjusted β coefficient	p-value
**Pneumonia type**		
CAP	Ref	
HAP	6.5 (4.0 – 9.0)	**<0.001**
VAP	11.9 (5.5 – 18.3)	**<0.001**
**Age**		
<60	Ref	
≥60	-0.1 (-2.1 – 1.9)	0.886
**Gender**		
Male	-1.1 (-3.0 – 0.9)	0.282
Female	Ref	
**Comorbidities**		
Hypertension	–	–
Diabetes mellitus	0.9 (-1.6 – 3.4)	0.482
Chronic lung disease	-2.2 (-5.2 – 0.8)	0.152
Chronic heart disease	–	–
Chronic liver disease	–	–
Chronic kidney disease	–	–
Malignancy	–	–
Pulmonary TB	-3.0 (-6.7 – 0.6)	0.102
Extrapulmonary TB	–	–
**Resistant pathogens**		
CR-Ab	7.4 (2.7 – 12.2)	**0.002**
CR-Kp	2.7 (-0.3 – 5.7)	0.082
DTR-Psa	7.7 (0.2 – 15.3)	**0.043**
MRSA	–	–
MRCoNS	–	–
**Interaction**		
**Pneumonia * Pulmonary TB**		
HAP	-4.9 (-15.7 – 6.0)	0.377
VAP	NA	NA
**Pneumonia * CR-Ab**		
HAP	-4.9 (-11.1 – 1.4)	0.126
VAP	-8.2 (-18.8 – 2.5)	0.132
**Pneumonia * DTR-Psa**		
HAP	5.8 (-3.5 – 15.0)	0.220
VAP	8.6 (-4.2 – 21.4)	0.186

CAP, community-acquired pneumonia; CR-Ab, carbapenem-resistant *Acinetobacter baumannii*; CR-Kp, carbapenem-resistant *Klebsiella pneumoniae*; DTR-Psa, difficult-to-treat *Pseudomonas aeruginosa*; HAP, hospital-acquired pneumonia; MRCoNS, methicillin-resistant coagulase negative *Staphylococcus*; MRSA, methicillin-resistant *Staphylococcus aureus*; VAP, ventilator-associated pneumonia.
'–' indicates variable not included in the final model; β coefficient indicates change in length of stay (days); 'ref' indicates the reference category; '*' are interaction terms.

Table S11. List of antibiotics tested.

Amikacin	Cefuroxime	Nitrofurantoin
Amoxicillin	Chloramphenicol	Oxacillin
Amoxycillin-Clavulanate	Ciprofloxacin	Penicillin G
Ampicillin	Clindamycin	Piperacillin
Ampicillin/sulbactam	Erythromycin	Piperacillin Tazobactam
Amphotericin B	Ertapenem	Quinupristin-dalfopristin
Aztreonam	Fluconazole	Rifampicin
Caspofungin	Flucytosine	Streptomycin
Cefazolin	Gentamicin	Teicoplanin
Cefepime	Imipenem	Tetracycline
Cefoxitin	Levofloxacin	Tigecycline
Cefotaxime	Linezolid	Trimethoprim/sulfamethoxazole
Ceftriaxone	Meropenem	Vancomycin
Ceftazidime	Micafungin	Voricanazole
Ceftazidime-clavulanic acid	Moxifloxacin	
